# 2,6-Bis(2-Benzimidazolyl)Pyridine (BBP) Is a Potent and Selective Inhibitor of Small Conductance Calcium-Activated Potassium (SK) Channels

**DOI:** 10.3389/fphar.2018.01409

**Published:** 2018-12-03

**Authors:** Rafel Simó-Vicens, Sofia H. Bomholtz, Ulrik S. Sørensen, Bo H. Bentzen

**Affiliations:** ^1^Cardiovascular Pharmacology, Department of Biomedical Sciences, Faculty of Health and Medical Sciences, University of Copenhagen, Copenhagen, Denmark; ^2^Acesion Pharma, Copenhagen, Denmark

**Keywords:** small conductance calcium-activated potassium channels, SK channels, K_Ca_2, BBP, 2, 6-bis(2-benzimidazolyl)pyridine, polycyclic pyridine, blocker

## Abstract

A variety of polycyclic pyridines have been proposed as inhibitors of the small conductance calcium-activated potassium (SK) channel. To this group belongs 2,6-bis(2-benzimidazolyl)pyridine (BBP), a commercially and readily available small organic compound which has earlier been described in a broad range of chemical and biological uses. Here, we show how BBP can also be used as a potent and specific SK channel blocker *in vitro*. The potency of BBP was measured using automatic patch clamp on all three SK channel subtypes, resulting in similar IC_50_ of 0.4 μM. We also assessed the selectivity of BBP on a panel of calcium-activated and voltage-activated potassium channels using two-electrode voltage clamp, automatic and manual patch clamp. BBP did not have any effect on IK, K_ir_2.1, K_ir_3.1+K_ir_3.4, K_v_1.5, K_v_4.3/K_CHIP_2 and K_v_7.1/KCNE1 currents and was 4.8-fold and 46-fold more potent on all SK channel subtypes vs. BK and hERG channels, respectively. Moreover, we were able to identify H491 as a critical amino acid for the pharmacological effect of BBP on the SK channel. From a medicinal chemistry perspective, BBP could be used as a starting point for the design of new and improved SK inhibitors.

## Introduction

Small conductance calcium-activated potassium (SK) channels are ion channels that open when the intracellular calcium concentration is increased, allowing the passage of potassium ions through cell membrane. These submicromolar changes in the intracellular calcium concentration are sensed by the protein calmodulin, which is covalently attached to the C-terminus of the channel (Adelman, 2016). The SK channel is now considered a promising novel target in a wide range of therapeutic areas such as cardiovascular (Gu et al., 2018), neurology (Kshatri et al., 2018), and oncology (Berthe et al., 2016). This interest has prompted the development of new molecules to both study and therapeutically target these channels. Since the discovery of apamin, a neurotoxin extracted from bee venom and the first specific SK inhibitor described (Habermann, 1984), the lists of both SK channel activators and inhibitors have grown (Weatherall et al., 2010; Christophersen and Wulff, 2015), including most recently the negative allosteric modulator AP14145 (Diness et al., 2017; Simó-Vicens et al., 2017a) and the natural flavone acacetin (Chen et al., 2017). However, some of these compounds may be difficult to access and/or use for a number of reasons such as low potency, incomplete pharmacological characterization or commercial unavailability.

**Figure 1:**
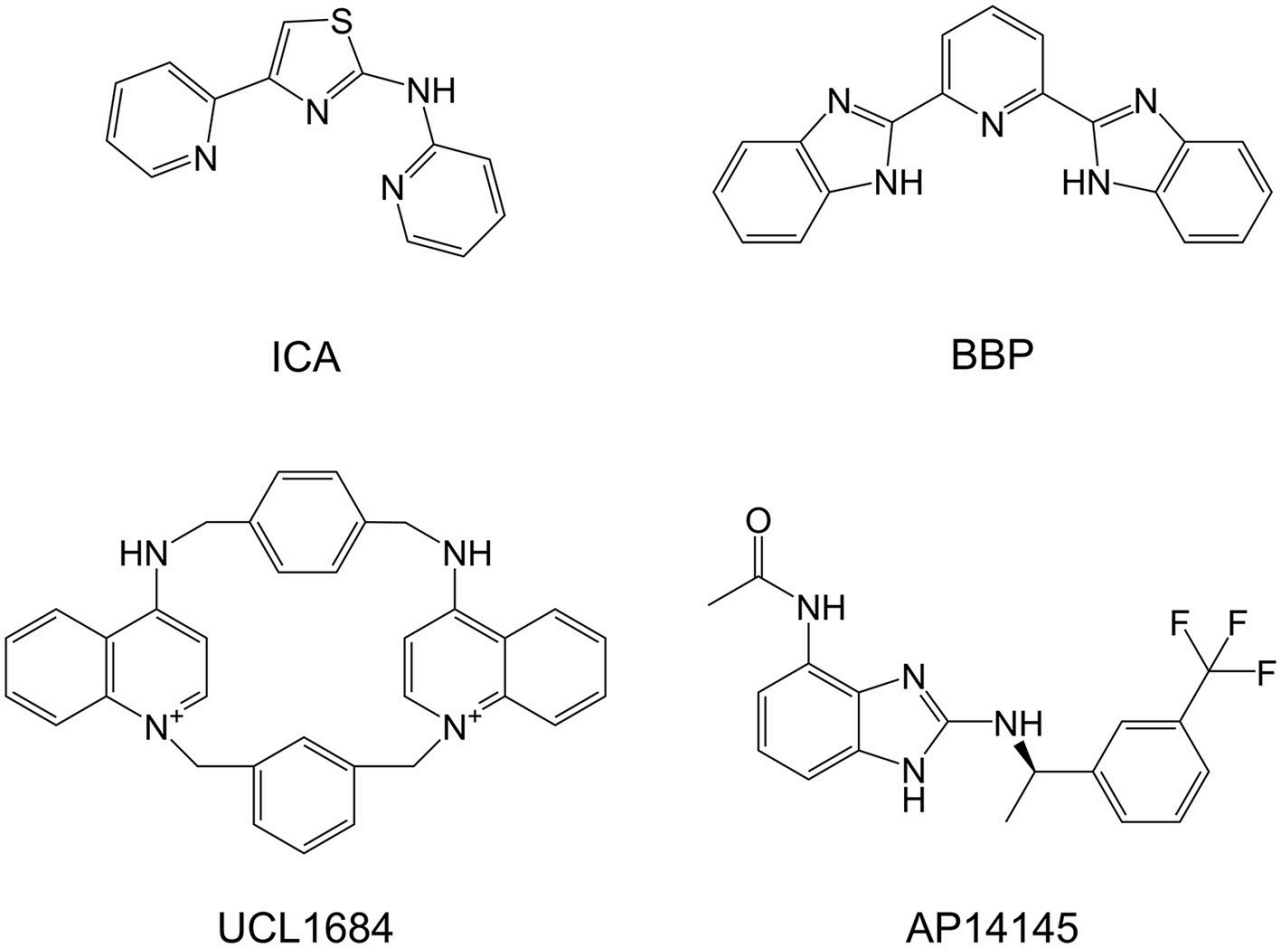
Chemical structures of SK blockers UCL1684 and ICA, SK negative allosteric modulator AP14145, and BBP.

In 2005, a patent application was filed proposing a variety of polycyclic pyridines as structurally related to neuromuscular inhibitors such as tubocurarine, pancuronium, and atracurium, and as having inhibitory effect on the SK channel, similarly to UCL compounds (Campos Rosa et al., 2000; Wang et al., 2005), a series of non-peptidic and potent SK inhibitors represented by UCL1684 (Figure 1). This patent includes *N*-(pyridin-2-yl)-4-(pyridin-2-yl)thiazol-2-amine (ICA, Figure 1), an apamin-displacing 2-aminothiazole (Gentles et al., 2008), which has not only proven to be a highly specific and potent inhibitor of SK channels but has also been successfully used in multiple studies as an antiarrhythmic agent (Diness et al., 2010; Skibsbye et al., 2014). The patent further describes a class of 2-benzimidazolyl pyridines, including 2,6-bis(2-benzimidazolyl)pyridine (BBP, Figure 1), a small organic compound which has in literature been described in a broad range of other chemical and biological uses, including as a chemosensor to detect toxic metabolites (Chetia and Iyer, 2006, 2007, 2008, 2011), as chemical catalyst in organic chemistry (Dai et al., 2017; Renuka and Gayathri, 2018) and as having pharmacological effects as potential antiviral (Musiu et al., 2014) and anticancer agent (Zheng et al., 2017). Most of these applications rely on the ability of BBP to mimic the biological histidine-imidazole system which can bind and accommodate metals and a number of organic molecules. Unfortunately, BBP was in the above patent application only generally described amongst many other compounds as an SK inhibitor without disclosing a specific IC_50_ and has never been properly pharmacologically characterized. Since BBP represents a readily available and interesting chemical scaffold for the development of new and improved SK inhibitors, we in this work aimed to thoroughly characterize and describe the potency and selectivity of BBP as an SK channel inhibitor.

Through different electrophysiological techniques, we have assessed the inhibitory potency of BBP on all three SK channel subtypes, as well as its selectivity on a panel of calcium-activated and voltage-activated potassium channels. Moreover, through mutagenesis studies, we have been able to identify the putative binding site of BBP.

## Materials and Methods

### Molecular Biology

rSK3 H491N was obtained through site-directed mutagenesis on the rSK3 WT gene (Department of Biomedical Sciences, Copenhagen, Denmark). This was achieved using the forward primer GAGTCTGTGAAAGGTACAATGACCAGCAGGACG and reverse primer CGTCCTGCTGGTCATTGTACCTTTCACAGACTC with the DNA polymerase PfuI Ultra II Fusion (Agilent, Santa Clara, California, United States). The resulting product was then used to transform competent *Escherichia coli* by thermic shock, which were plated on agar plates containing the corresponding selection antibiotic and incubated overnight at 37°C. The next day, plasmid DNA was purified using standard methods and sent for sequencing to verify the construct.

HEK293 cells were transiently co-transfected with the plasmid of interest (hBK, hSK1, hSK2, hSK3 WT, rSK3 H491N, or hIK) and eGFP using standard Lipofectamine™ 2000 (Thermo Fisher Scientific, Waltham, Massachussets, USA) transfection protocols. After transfection, cells were incubated overnight before the experiments.

### Cell Lines Culture

Three different stable HEK293 cell lines expressing hSK1, hSK2 or hSK3 (NeuroSearch A/S, Ballerup, Denmark) and a CHO stable cell line expressing hERG (University of Copenhagen, Copenhagen, Denmark) were used for automated patch clamp. These cells were cultivated and prepared as previously described (Skarsfeldt et al., 2016).

WT HEK293 cells used for transient transfections were cultured in the same way as HEK293 stable cell lines in the absence of the selection antibiotic geneticin.

### Chemicals

2,6-Bis(2-benzimidazolyl)pyridine (BBP), dofetilide, bicuculline methiodide, paxilline, 1-[(2-chlorophenyl)diphenylmethyl]-1*H*-pyrazole (TRAM-34) and 4-aminopyridine were purchased from Sigma-Aldrich (Germany). 2-(4-Chlorophenoxy)-2-methyl-*N*-[5-[(methylsulfonyl)amino]tricyclo[3.3.1.13,7]dec-2-yl]-propanamide (JNJ 303) was purchased from Tocris (United Kingdom) and *N*-(2-{[(1*R*)-1-[3-(trifluoromethyl)phenyl]ethyl]amino}-1*H*-1,3-benzodiazol-4-yl)acetamide (AP14145) was synthetized by Syngene Inc. (Bangalore, India). All compounds were dissolved in pure dimethyl sulfoxide (Sigma-Aldrich, Germany) into 10 mM stock solutions and stored at −20°C. On the day of the experiment, the stock solution was thawed and the corresponding amount of compound was added to the working solution.

### Electrophysiology

#### Automated Patch-Clamp

Automated patch clamp (QPatch 16, Sophion, Denmark) on SK-expressing HEK293 cell lines was conducted as previously described (Simó-Vicens et al., 2017b). Briefly, SK currents were elicited by voltage-ramps starting at −80 mV and finishing at +80 mV from a holding potential of 0 mV. Extracellular solution contained 0.1 mM CaCl_2_, 3 mM MgCl_2_, 154 mM KCl, 10 mM HEPES and 10 mM glucose with pH 7.4. Intracellular solution for SK experiments contained 8.106 mM CaCl_2_ (final free calcium concentration of 400 nM), 1.167 mM MgCl_2_, 10 mM EGTA, 154 mM KCl, 10 mM HEPES, 31.25/ 10 mM KOH/EGTA and 15 mM KOH with pH 7.2. Automated patch clamp on hERG-expressing CHO cell line was performed also as previously described (Skarsfeldt et al., 2016).

#### Manual Patch-Clamp

Manual patch clamp experiments were performed at room temperature in the whole cell configuration with a HEKA EPC9 amplifier and the Patchmaster software (HEKA Elektronik, Ludwigshafen, Germany). All currents were elicited every 2 s using an incremental 200 ms voltage-ramp protocol starting at −80 mV and finishing at +80 mV from a holding potential of 0 mV. Borosilicate glass pipettes were pulled with a horizontal puller (DMZ Universal Puller, Zeitz Instruments, Martinsried, Germany) with resistances 2.5–3 MΩ. Currents were measured using the same solutions as the ones used for automated patch clamp, with symmetrical potassium concentrations and a free intracellular calcium concentration of 400 nM. In hBK experiments, intracellular solution was modified as previously described (Strøbæk et al., 2006) and free intracellular calcium concentration was reduced to 100 nM. On the day of the experiment, cells were rinsed with magnesium-free PBS and detached with Detachin™ (Amsbio, Abingdon, United Kingdom) before being plated on 0.5 mm diameter coverslips. Only fluorescent cells were considered for patch clamp experiments.

#### Two-Electrode Voltage Clamp (TEVC)

cRNA was prepared from linearized plasmids using the mMESSAGE mMACHINE T7 kit (Ambion, USA) and quantified by UV spectroscopy (NanoDrop, Thermo Scientific, Wilmington, USA). *Xenopus laevis* oocytes were acquired from EcoCyte Bioscience (Castrop-Rauxel, Germany). Oocytes were injected with 50 nL cRNA and currents were recorded after 2 days of incubation at 19°C. Currents were recorded at a sampling rate of 10 kHz using a TEVC amplifier (Dagan CA-1B; IL, USA), and borosilicate glass recording electrodes (Module Ohm, Herlev, Denmark), made with a DMZ Universal Puller (Zeitz Instruments, Martinsried, Germany) with a resistance of 0.5–1 MΩ when filled with 2 M KCl. Oocytes were superfused with Kulori (pH 7.4) solution containing NaCl 90 mM, KCl 4 mM, MgCl_2_ 1 mM, CaCl_2_ 1 mM, 4-(2-hydroxyethyl)-1-piperazineethanesulfonic acid 5 mM, and currents were elicited with the voltage protocols previously indicated (Skarsfeldt et al., 2016), from a holding potential of −80 mV. Data acquisition was performed with the Patchmaster software (HEKA Elektronik, Ludwigshafen, Germany).

### Data Analysis

All data were analyzed using GraphPad Prism 7 and results are presented as mean ± SEM. Statistical differences are considered significant when *p* < 0.05. *n* refers to the number of cells that have been tested for each experiment.

Individual IC_50_ values were calculated for each cell with the built-in nonlinear fit log(inhibitor) vs. normalized response equation:

(1) $$\begin{array}{r} Y=\frac{100}{1+10^{X-logIC50}} \end{array}$$

Where *Y* is the normalized response, from 100, corresponding to current recorded during saline perfusion, down to 0, corresponding to currents recorded in the presence of bicuculline methiodide 100 μM. *X* is the log of the BBP concentration. A standard Hill Slope of −1 was considered for this purpose. The resulting IC_50_ were then used in one-way ANOVA statistical analysis to detect potential subtype selectivity among hSK1, hSK2, and hSK3. Finally, individual IC_50_ values were used to calculate the total mean ± SEM.

To assess the effect of BBP on other calcium-activated potassium channels (hBK, hSK3 WT, rSK3 H491N, and hIK), each individual current was first normalized to its cell size using membrane's capacitance (pA/pF) before statistical analysis. For the rest of voltage-activated potassium channels (K_ir_2.1, K_ir_3.1+K_ir_3.4, K_v_1.5, K_v_4.3/K_CHIP_2, K_v_7.1/KCNE1 and hERG), raw data from current measurements were directly used for statistical analysis. Statistical significance between saline and the effect of both BBP and the corresponding reference compound was determined for each channel using one-way ANOVA analysis. To compare the effect of BBP on hSK3 WT and rSK3 H491N, individual raw data for each cell were first normalized. In this case, currents elicited under saline perfusion were considered as full channel activation and currents elicited with the presence of either bicuculline methiodide (100 μM) or AP14145 (10 μM) were considered as full channel inhibition. Later, the resulting data were compared using unpaired *t*-test to determine statistically significant differences of inhibitory effect of BBP between the WT and mutant channels. For graphic and comparative purposes in Figure **5**, corresponding raw data were normalized using current values under saline perfusion as fully activated and the value 0 as fully inhibited currents. These normalized data were not used in statistical analysis.

## Results

### BBP Inhibits All SK Channel Subtypes With Similar Potency

**Figure 2:**
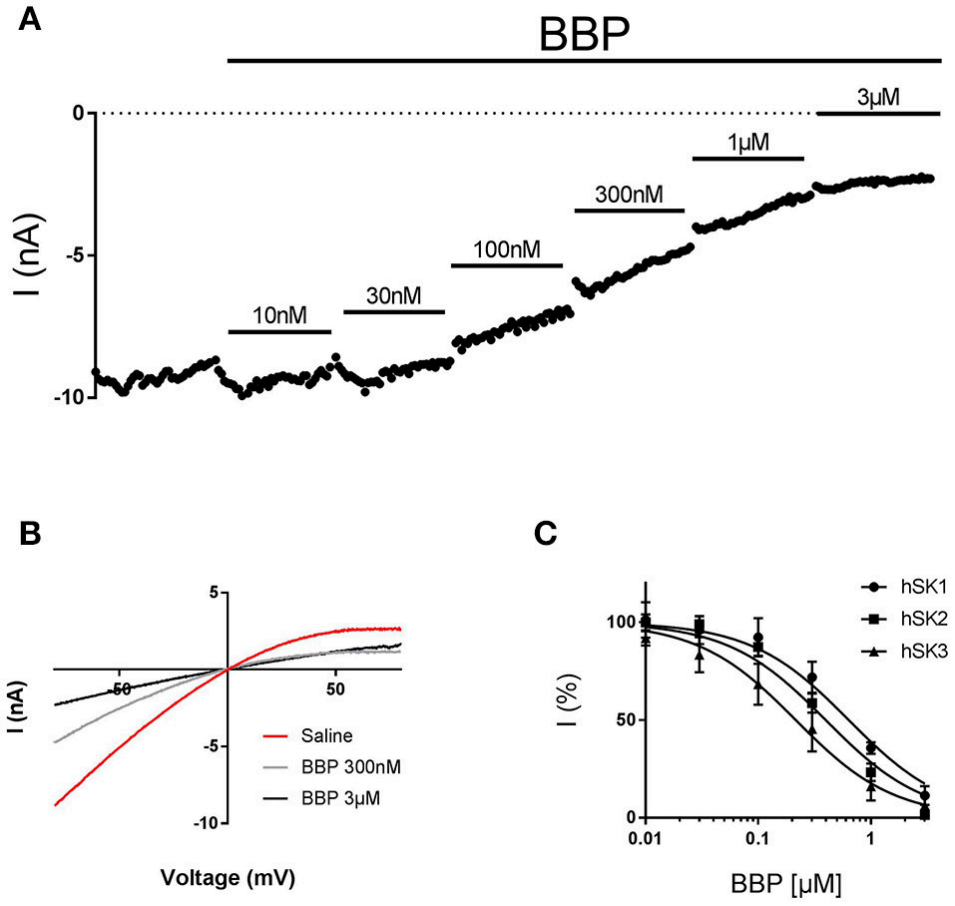
Inhibitory effect of BBP on hSK channels. Representative current-time plot **(A)** and its corresponding current-voltage plot **(B)** from automated patch clamp experiments picturing the inhibitory effect of BBP at different increasing concentrations performed on hSK3-expressing cells. The compound completely inhibited SK currents at concentrations of 1–3 μM. **(C)** Depicts the inhibition curves of BBP on each of the hSK subtype channels Resulting IC_50_ were 0.7 ± 0.2 μM for hSK1 (*n* = 9), 0.4 ± 0.1 μM for hSK2 (*n* = 8) and 0.4 ± 0.2 μM for hSK3 (*n* = 9).

To assess the *in vitro* potency and potentially subtype selectivity of BBP, we used the automated patch clamp platform QPatch 16. These experiments were conducted on the whole cell configuration, using three different stable HEK cell lines expressing hSK1, hSK2, or hSK3. During the experiments, each cell was exposed to six increasing concentrations of BBP, starting at 10 nM and up to 3 μM. In the end, bicuculline methiodide 100 μM, which was used as positive control and reference, was added. All three hSK subtypes were inhibited by BBP. hSK currents were inhibited in a dose-dependent manner, starting at submicromolar concentrations and reaching total inhibition at 3 μM (Figures 2A,B). These data were used to calculate BBP IC_50_ on each of the hSK channel subtypes resulting in 0.7 ± 0.2 μM for hSK1 (n = 9), 0.4 ± 0.1 μM for hSK2 (n = 8) and 0.4 ± 0.2 μM for hSK3 (n = 9, Figure 2C). No significant statistical differences were found between IC_50_ values, suggesting that BBP is equipotent on all SK subtypes.

### Effect of BBP on Other Calcium-Activated Potassium Channels

After testing BBP on the three SK channel subtypes, we decided to explore whether other calcium-activated potassium channels were affected by the compound. To do so, we transiently transfected HEK293 WT cells with a plasmid containing one of the human SK channel subtypes, the big conductance calcium and voltage-activated potassium (BK) channel or the intermediate conductance calcium-activated potassium (IK) channel. These cells were plated on glass coverslips and used for whole cell manual patch clamp experiments. The cells were first perfused with saline solution until a stable baseline was reached. The solution in the bath was thereafter changed, and the cells were exposed to BBP (1 μM) which was perfused for at least 2 min or until full effect of the drug was reached. Finally, a positive control was used to inhibit the remaining current and quantify the total effect of BBP. Both hBK and hIK currents were elicited with the same voltage ramp protocol as the one described for hSK currents.

First, we wanted to confirm that 1 μM BBP was sufficient to completely block all SK channel subtypes on the manual patch clamp setup. BBP 1 μM inhibited 93.7 ± 1.6% of hSK1 current (*n* = 6), 94.8 ± 2.1% of hSK2 current (*n* = 5), and 92.5 ± 2.3% of hSK3 current (*n* = 5) when compared to the positive control bicuculline methiodide (100 μM). These results confirm the equipotency of BBP among all SK channel subtypes as previously observed on the automated patch clamp. Moreover, in order to assess possible voltage-dependent inhibition of BBP, we compared its effect at −80 and +80 mV. Statistical analyses showed no significant differences between inhibition at −80 or +80 mV.

**Figure 3:**
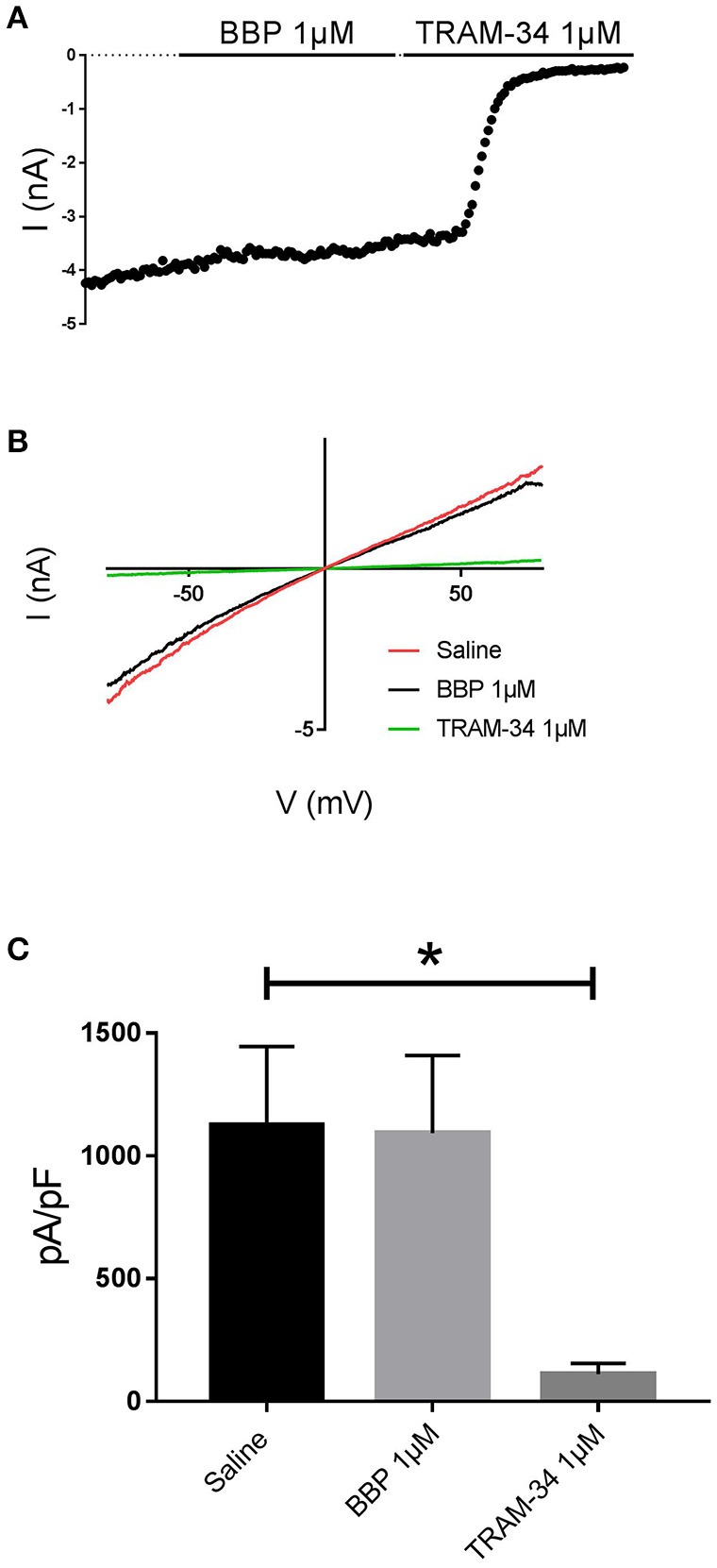
Effect of BBP 1 μM on hIK (*n* = 6). Representative current-time plot **(A)** and its corresponding current-voltage plot **(B)** depicting the effect of BBP 1 μM and the specific IK inhibitor TRAM-34 1 μM. **(C)**, a column graph compares the effects of both BBP and TRAM-34 with the control current. IK current was not affected by BBP 1 μM. *p < 0.05.

**Figure 4:**
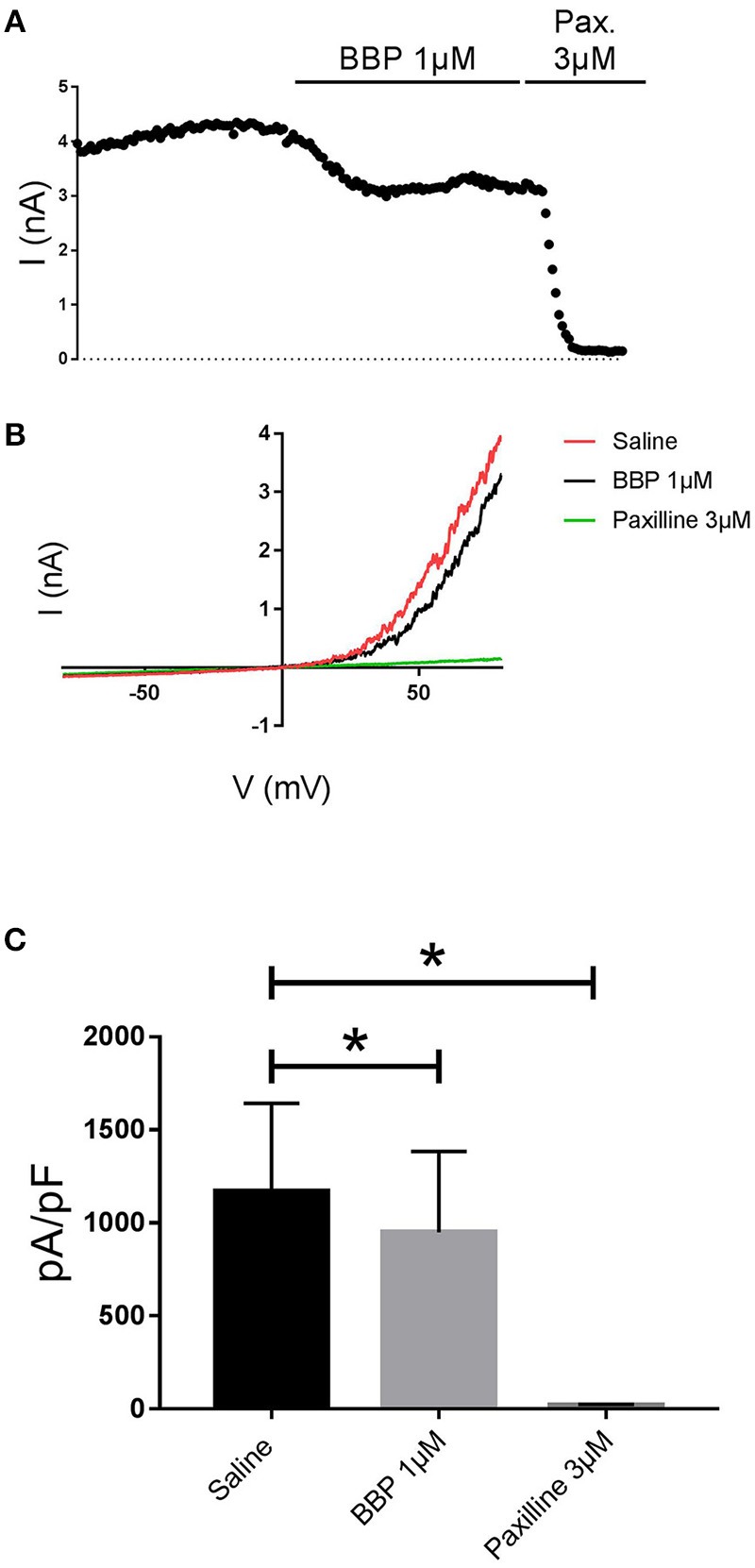
Effect of BBP 1 μM on hBK (*n* = 8). Representative current-time plot **(A)** and its corresponding current-voltage plot **(B)** depicting the effect of BBP 1 μM and the specific BK inhibitor paxilline 3 μM. The column graph **(C)** compares the effects of both BBP and paxilline with the control current. BK current was inhibited by 19 ± 6% upon application of BBP 1 μM. *p < 0.05.

When BBP (1 μM) was applied on HEK293 cells transfected with hIK, the elicited current remained unaffected (*n* = 6, Figures 3A,B). The same IK current was completely inhibited by the specific blocker TRAM-34 1μM (Wulff et al., 2000, Figure 3C). In contrast, hBK transfected cells were only partially but significantly inhibited by BBP 1 μM (*n* = 8, Figures 4A,B). Inhibition by BBP resulted in a 19 ± 6% reduction of the hBK current. The remaining current was totally inhibited after the cell was exposed to paxilline 3μM (Figure 4C), a specific negative BK channel modulator (Sanchez and McManus, 1996).

**Figure 5:**
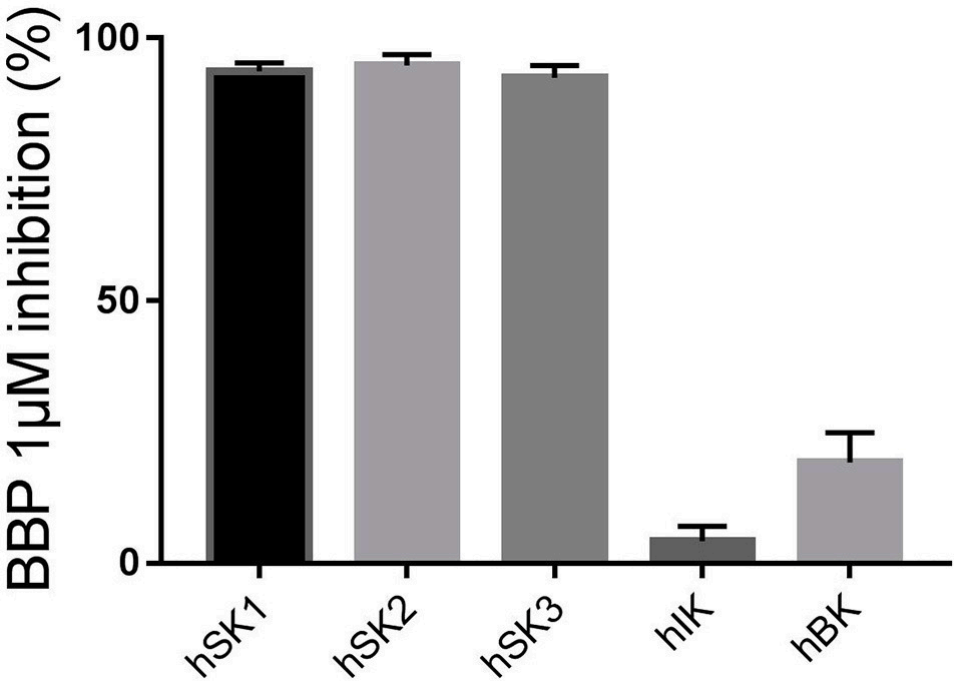
Column graph comparing the inhibitory effect of BBP 1 μM measured on the manual patch clamp on all calcium-activated potassium channels: SK1 (*n* = 6), SK2 (*n* = 5), SK3 (*n* = 5), IK (*n* = 6) and BK (*n* = 8) channels. BBP 1 μM totally inhibited all SK currents as well as 19 ± 6% of BK current. However, the IK channel was not affected by BBP 1 μM.

The inhibitory effect of BBP 1 μM on all calcium-activated potassium channels has been combined in Figure 5.

### Effect of BBP on Other Potassium Channels

**Figure 6:**
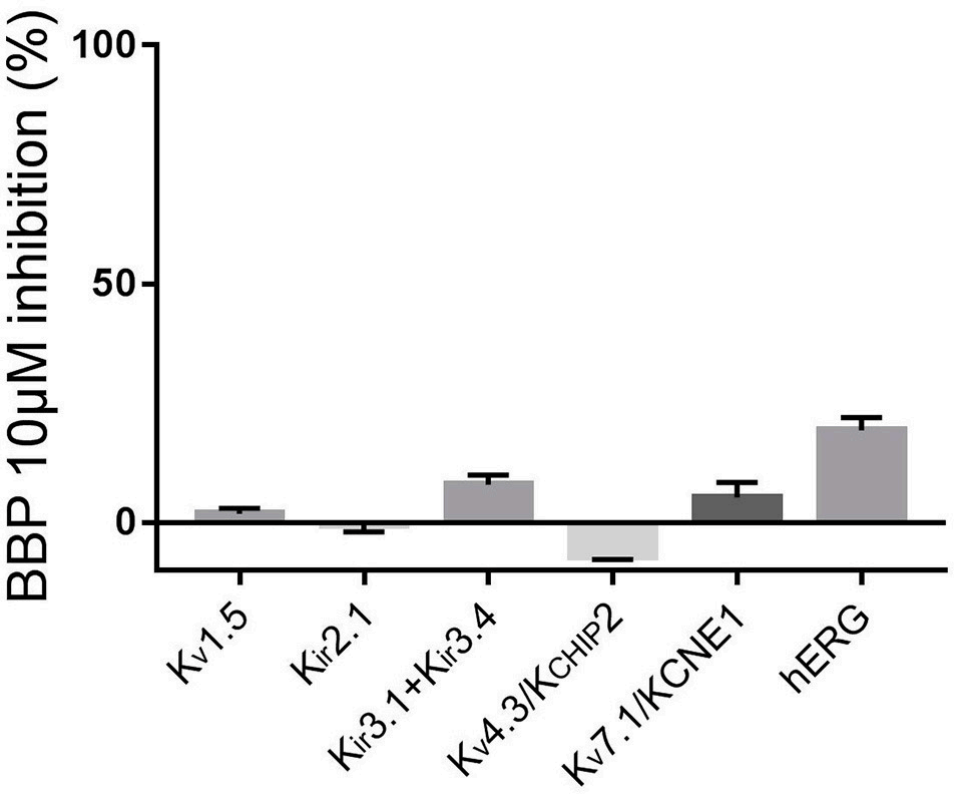
Column graph comparing the inhibitory effect of BBP 10 μM on a panel of voltage-gated potassium channels (*n* = 5 for each current). Except hERG, none of the tested currents were significantly affected by the compound.

To complete the selectivity profile of BBP, we tested the drug on the following potassium channels: inward rectifying potassium channels K_ir_2.1 and K_ir_3.1+K_ir_3.4, voltage-gated potassium channels K_v_1.5, K_v_4.3/K_CHIP_2 and K_v_7.1/KCNE1 and the ether-à-go-go-related-gene potassium channel hERG. Except for hERG, all channels were expressed in oocytes from *Xenopus laevis* previously injected with the corresponding cRNA. Two days after the injection, the oocytes were ready for TEVC experiments. First, the oocytes were perfused with Kulori solution. After 3 min, when stable current levels were achieved, the solution was changed to a Kulori solution containing BBP 10 μM and the oocyte superfused for 3 additional minutes or until full drug effect was achieved. Finally, the remaining current was inhibited with a reference inhibitor used as positive control: Ba^2+^ 100 μM for K_ir_2.1, terteapine-Q 1 μM for K_ir_3.1+K_ir_3.4, 4-aminopyridine 4 mM for both K_v_1.5 and K_v_4.3/K_CHIP_2, and finally JNJ303 1 μM for K_v_7.1/KCNE1. Application of BBP 10 μM did not significantly affect any of the aforementioned currents (*n* = 5 for each current, Figure 6).

Effects on hERG were tested using automated patch clamp on a stable CHO cell line expressing the hERG channel. In this case, we used dofetilide 500 nM as reference. This time, when cells were exposed to BBP 10 μM, hERG current was reduced by 19 ± 3% (*n* = 5, Figure 6).

### H491 Is Critical for BBP Inhibition on rSK3

**Figure 7:**
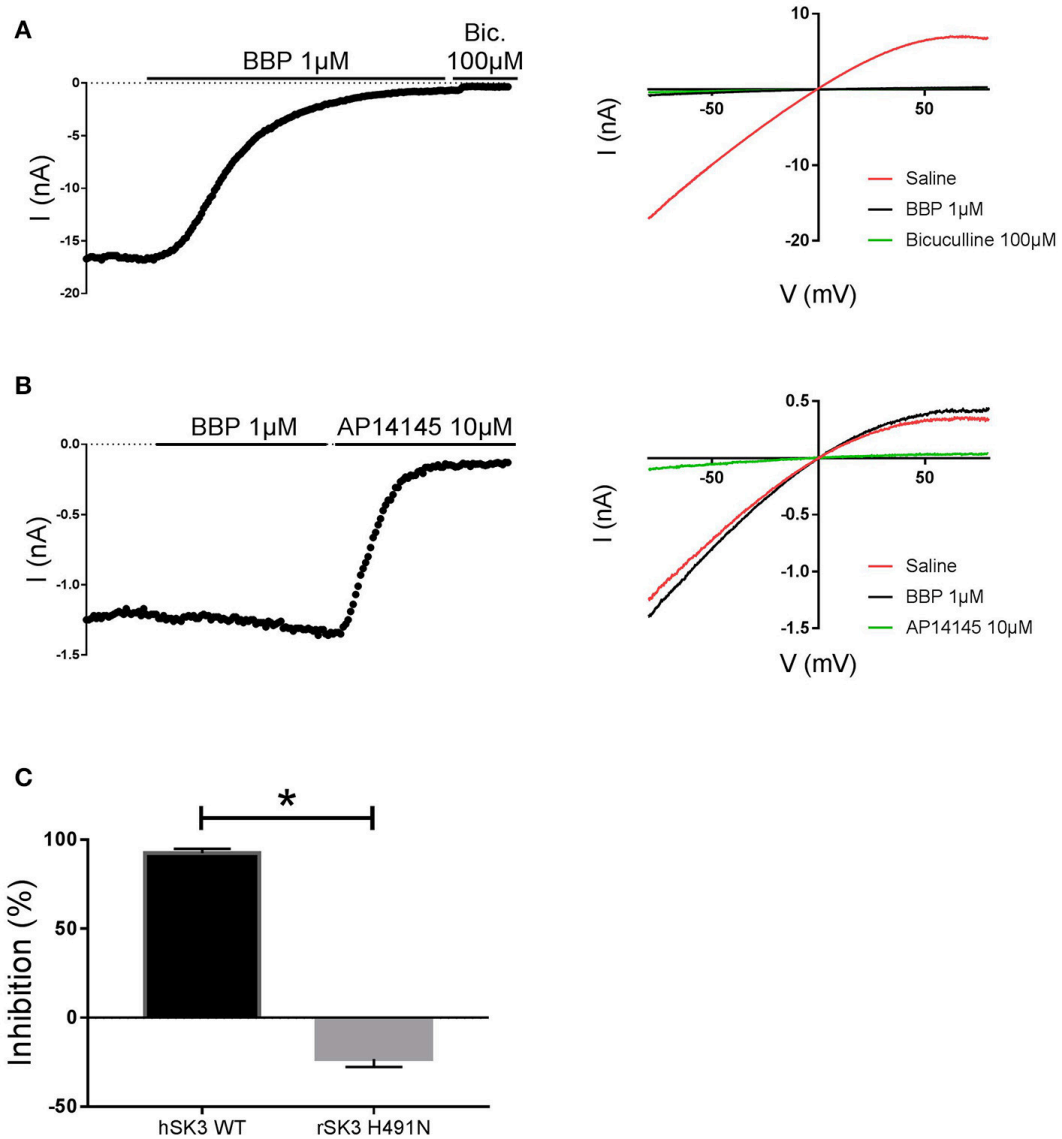
Effect of BBP 1 μM on hSK3 WT (*n* = 5) and rSK3 H491N (*n* = 8). Representative current-time plots (left) and their corresponding current-voltage plots (right) depicting the effect of BBP 1 μM on hSK3 WT **(A)** and rSK3 H491N **(B)**. The results are summarized in the column graph **(C)** comparing the effect of the compound on both the WT and mutant channel. The mutant rSK3 H491N channel was insensitive to the inhibitory effect of BBP compared to the WT channel. *p < 0.05.

In an attempt to elucidate possible binding determinants for BBP, we decided to test the drug on the apamin-insensitive rSK3 H491N mutant, an amino acid located at the extracellular loop between S5 and the P-loop of the channel (Lamy et al., 2010). To this end, we transiently transfected HEK cells with rSK3 H491N mutant and used these to perform whole cell manual patch clamp experiments. Again, currents were elicited by voltage ramps ranging from −80 to +80 mV, from a holding potential of 0 mV, and channels were activated with 400 nM of free intracellular calcium. Once the mutant SK current was stable, BBP 1 μM was applied on the cell for at least 2 min or until the drug reached its total effect. At the end of the experiment, AP14145 10 μM was added to the bath and used as reference. Surprisingly, and in contrast to hSK3 WT (Figure 7A), rSK3 H491N current was not inhibited by BBP 1 μM (*n* = 8), but it was slightly increased in the presence of the drug (Figures 7B,C).

## Discussion

SK channels have gained increasing interest during recent years, being considered as promising therapeutic targets for multiple diseases through a wide spectrum of therapeutic areas (Berthe et al., 2016; Gu et al., 2018; Kshatri et al., 2018). Since the discovery of the first specific SK inhibitor, apamin (Habermann, 1984), research efforts have been invested in the development and discovery of new molecules targeting SK channels. This has resulted in a gradually and expanding list of both SK inhibitors and activators, ranging from synthetic and natural peptides to small molecules of a variety of chemical classes and including different mechanisms of action (Weatherall et al., 2010; Christophersen and Wulff, 2015). Despite this progress, many of the disclosed molecules may not be suitable as practical tool compounds for the study of SK channels. From an accessibility perspective these compounds may not be commercially available and only achievable in small amounts. In addition, compounds may not be pharmacologically well characterized and with sufficient SK channel potency or general selectivity. Moreover, expansion of SK channels research, has further challenged currently available pharmacological tools which have been confronted with insensitive heteromeric channels (Hancock et al., 2015), difficult to access intracellular locations (Krabbendam et al., 2018), unexpected inhibitory effects (Voos et al., 2017), and intracellular calcium dependence (Strøbæk et al., 2006; Simó-Vicens et al., 2017a).

In the past, a series of 2-benzimidazolyl pyridines, including 2,6-bis(2-benzimidazolyl)pyridine (BBP), were claimed in a patent as being SK channel inhibitors (Wang et al., 2005). As the pharmacological data disclosed was very general we decided to characterize BBP on our automated patch clamp platform to assess its potency on all SK channel subtypes. These experiments revealed that BBP was able to inhibit the three SK channel subtypes at submicromolar concentrations (Figures 2A,B), with similar potency and an IC_50_ of ~0.4 μM (Figure 2C). Because BBP is an interesting and versatile chemical scaffold we thought that it could be potentially used as a new starting building block for the development of new and improved SK blockers. We therefore expanded the pharmacological characterization and assessed the selectivity of BBP over a panel of calcium and voltage-activated potassium channels. Within the calcium-activated potassium channels, it was found that the IK channel was not affected by BBP 1 μM (Figures 3A,B) whereas BK channel was inhibited by 19 ± 6% (Figure 4C), an inhibitory effect 4.8-fold lower than on any SK currents. Interestingly, this relation between BK and SK channels has also been observed for the allosteric modulator AP14145 (Simó-Vicens et al., 2017a). In the case of voltage-activated potassium channels, none of the six tested currents (K_ir_2.1, K_ir_3.1+K_ir_3.4, K_v_1.5, K_v_4.3/K_CHIP_2, K_v_7.1/KCNE1, hERG) were significantly affected by BBP 10 μM, except for hERG, which was reduced by 19 ± 3% (Figure 6). Although the present work shows a promising selectivity profile of BBP over eight calcium and voltage-activated potassium channels, further selectivity profiling over different ion channels and proteins as well as pharmacokinetic assessments will be needed to determine the suitability of BBP for each specific application.

In addition to the pharmacological profiling, and to get a better understanding of the mechanism of action of BBP on the SK channel, we conducted a study to possibly identify important molecular determinants for BBP through mutagenesis studies. To this end, we used the rSK3 H491N mutant which is known to be insensitive to apamin, _D_-tubocurarine and UCL1684 (Lamy et al., 2010), all being potent SK channel blockers. Interestingly, the inhibitory effect of BBP was completely abolished by the mutation (Figure 6B), suggesting that the compound binds to the extracellular loop between S5 and the P-loop of the channel, sharing its binding site with apamin. In contrast to the allosteric SK channel modulators NS8593 (Strøbæk et al., 2006; Jenkins et al., 2011) and AP14145 (Simó-Vicens et al., 2017a), BBP thus appears to have common mechanism with compounds earlier claimed to block the pore of the ion channel, such as UCL1684 and ICA. Here it is interesting to note that the patent mentioning ICA and BBP (Wang et al., 2005) also describes this class of compounds as having ability to form a complex with polyvalent metal cations. This is suggested as a tridentate interaction between the metal cation and three nitrogen atoms comprised in the core and the connected heterocycles of the SK inhibitor, in BBP being the central pyridine and a nitrogen of each of the two benzimidazole moieties (Figure 1). Any presence or influence of such metal complex in the mechanism-of-action of BBP and ICA has not been studied or described to date and further studies are needed to fully understand the mechanism of action of this class of SK inhibitors and its relation to other described inhibitors.

In summary, BBP is a potent and selective SK inhibitor with pharmacological action critically depending on interaction with histidine residue H491. BBP is readily available and together with the herein described selective profile we envision that it can become a new versatile and interesting starting block for the development of future SK channel blockers.

## Author Contributions

RS-V: conception and design of the work, automatic and manual patch clamp data acquisition and analysis, molecular biology and drafting of the manuscript. SB: automated patch clamp data acquisition and analysis, critical revision of the manuscript. US: conception and design of the work, critical revision of the manuscript. BB: conception and design of the work, interpretation of the data, drafting and critical revision of the manuscript.

### Conflict of Interest Statement

All authors are employed by Acesion Pharma.
